# Signatures of selection detected from whole‐genome sequencing indicate that the small body size in dwarf rabbit breeds is caused by polygenic effects with a few major loci

**DOI:** 10.1111/age.70025

**Published:** 2025-07-03

**Authors:** Samuele Bovo, Miguel Carneiro, Anisa Ribani, Matteo Bolner, Valeria Taurisano, Giuseppina Schiavo, Michele Schiavitto, Francesca Bertolini, Luca Fontanesi

**Affiliations:** ^1^ Animal and Food Genomics Group, Division of Animal Sciences, Department of Agricultural and Food Sciences University of Bologna Bologna Italy; ^2^ BIOPOLIS Program in Genomics, Biodiversity and Land Planning CIBIO Vairão Portugal; ^3^ CIBIO, Centro de Investigação Em Biodiversidade e Recursos Genéticos, InBIO Laboratório Associado Universidade do Porto Vairão Portugal; ^4^ Associazione Nazionale Coniglicoltori Italiani (ANCI) Contrada Giancola Snc Volturara Appula Italy

**Keywords:** DNA‐pool sequencing, dwarfism, *Oryctolagus cuniculus*, polymorphism, selection sweep

## Abstract

Early genetic studies have suggested that body size in rabbits can be considered a quantitative trait. Several rabbit breeds can be distinguished based on body size, including a few dwarf breeds differentiated by other morphological characteristics. While a large deletion in the *HMGA2* gene is a major locus associated with dwarfism in Netherland Dwarf rabbits, it may not fully explain the reduced body size in this breed or other dwarf breeds. In this study, we compared the genomes of two dwarf rabbit breeds (Dwarf Lop and Netherland Dwarf) with those of non‐dwarf rabbits by analysing whole‐genome sequencing data obtained using a DNA‐pool sequencing approach. We applied the fixation index (*F*_ST_) and pooled heterozygosity (*H*_P_) statistics to identify signatures of selection related to small body size by contrasting dwarf with non‐dwarf breeds and comparing dwarf breeds. We identified several genomic regions that contain genes previously linked to body dimensions in various species, including *LCORL*‐*NCAPG*, *COL2A1*, *GHRHR* and *CENPE*. Functional enrichment analysis of genes within the top differentiated regions revealed biological terms related to skeletal development, further supporting the biological relevance of these loci. Additionally, the use of the latest version of the reference rabbit genome enabled the identification of a genomic region containing *FGFR3*, a gene linked to achondroplasia. Some genomic regions showed differentiation between the two dwarf breeds, suggesting that their small body size may, in part, arise through different genetic mechanisms. Overall, these findings support a polygenic architecture underlying small size in rabbits, influenced by a few major loci.

## INTRODUCTION

The domestication process of the European rabbit (*Oryctolagus cuniculus*), commonly referred to as the domestic rabbit or simply rabbit, probably began in the High Middle Ages in the South of France, from wild rabbit populations belonging to the *O. c. cuniculus* subspecies (reviewed in Ferrand, 2008 and in Fontanesi et al., 2021; Carneiro et al., 2014). Over the following centuries, the dispersion and transfer of rabbits across Europe led to the development of numerous breeds, a process that accelerated significantly at the end of the nineteenth century and continued throughout the twentieth century (Fontanesi, 2021a). Most modern rabbit breeds can be distinguished by exterior traits, such as colour and morphological traits, and are often named after their colouration and body size. Recently constituted rabbit breeds were derived from crossbreeding activities of fancy breeders who created new combinations of colourations and morphological features by introgressing preexisting varieties and morphs into new lines or strains (Fontanesi, 2021a; Whitman, 2004).

In terms of adult body weight, rabbit breeds are traditionally classified into four major categories: dwarf, small, medium and large, with a gradient going from <1.0 kg to >5.0 kg (Boucher et al., 2021). Dwarf and small breeds cannot always be separated as variability within breeds may be large and partially overlapping (Ballan et al., 2022; Boucher et al., 2021; Whitman, 2004). The first studies that investigated the genetic basis of size inheritance in rabbits were published at the beginning of the twentieth century (Castle, 1914; MacDowell, 1914a, 1914b; Wright, 1918) using controlled crosses between large and small rabbit strains. Size inheritance was also studied by Castle (1922) by measuring the length of long leg bones and dimensions of the skull, body weight and ear length. Other early studies were mainly based on weight or growth rate as the primary criterion of size (Castle, 1929; Pease, 1928; Punnett & Bailey, 1918). Most of these studies suggested a polygenic mode of inheritance for body size and weight, with a few major loci containing recessive or partially dominant alleles determining some forms of monogenic dwarfism (reviewed in Fontanesi, 2021b).

The rabbit *Dwarf* locus was initially described by Greene et al. (1934) and subsequently by other authors (Brown & Pearce, 1945; Castle & Sawin, 1941; Greene, 1940; Latimer & Sawin, 1955a, 1955b, 1955c, 1963; Nachtsheim, 1937). The mutated allele (*dw*) is lethal in homozygous condition (*dw*/*dw*). Newborn rabbits with this genotype, known as peanuts, are smaller than their litter mates with the *Dw*/*dw* genotype, exhibit a swollen head, tiny ears, and usually die within a few days after birth. Heterozygous *Dw*/*dw* rabbits typically reach about two‐thirds the size of homozygous normal (*Dw*/*Dw*) rabbits and have compact, rounded bodies, a disproportionately larger head compared with the rest of the body, small ears, and a short snout owing to altered craniofacial development. These characteristics define a series of dwarf rabbit breeds known by various names in different countries, including Netherland Dwarf, Coloured Dwarf and many others. Carneiro et al. (2017) reported that the causal mutation of the *dw* allele in Netherland Dwarf rabbits is a 12.1 kb deletion in the *high mobility group AT‐hook 2* (*HMGA2*) gene on rabbit chromosome (OCU) 4, which results in the loss of the promoter and the first three exons. However, a recent study found that Netherland Dwarf rabbits from a Vietnamese population are not always heterozygous for the deleted *HMGA2* allele, with 34% being homozygous for the normal allele (Nguyen et al., 2024). Other signatures of selection across the genome were identified when comparing genomic sequencing data from Netherland Dwarf rabbits to sequencing data from non‐dwarf breeds (Carneiro et al., 2017). In a study of genome‐wide signature of selection based on high‐density single nucleotide polymorphisms, which included the Coloured Dwarf breed (similar to the Netherland Dwarf breed), Dwarf Lop breed, Ermine breed (a small‐sized breed) and 12 other normal‐sized or giant breeds (Ballan et al., 2022), several genomic regions were identified that may distinguish dwarf/small breeds from non‐dwarf breeds. Some of these regions were previously reported by Carneiro et al. (2017), in addition to several others.

**FIGURE 1:**
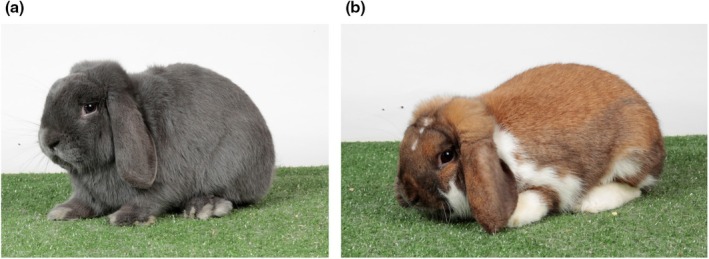
Dwarf Lop rabbits. (a) Blue colour. (b) Madagascar colour.

In this study, we generated whole‐genome sequencing data from Dwarf Lop rabbits (belonging to a breed with a different craniofacial structure than that of Netherland Dwarf rabbits; see Figure 1), and combined them with genomic information from Netherland Dwarf rabbits and other non‐dwarf breeds. By comparing these datasets, we identified signatures of selection that provide evidence for the polygenic determination of small size in rabbits, with a few major loci.

## MATERIALS AND METHODS

### Dwarf Lop rabbits, DNA samples, genotyping for the *HMGA2* deletion and sequencing

Hair roots were sampled from 14 Dwarf Lop rabbits (Figure 1). The animals were selected from the national Herd Book maintained by the Italian Rabbit Breeders Association (ANCI) based on the following criteria: (i) they exhibited standard breed characteristics; and (ii) they were not closely related (no full‐ or half‐sibs) and therefore can be considered a random sample for this breed population. The rabbits were not specifically bred, raised, or treated for this study; therefore, no ethical concerns are applicable.

DNA was extracted using the Wizard Genomic DNA Purification kit (Promega Corporation, Madison, WI, USA). The Dwarf Lop rabbits were then genotyped for the *HMGA2* deletion described by Carneiro et al. (2017). For this analysis, two PCR primer pairs were used in a multiplex PCR assay. Details on the primer sequences and PCR reactions are given in Table S1 (Carneiro et al., 2017). Whole‐genome sequencing information from the Dwarf Lop rabbits was obtained using a DNA‐pool sequencing approach. The DNA pool was created with an equimolar concentration of DNA from each of the 14 individuals included in the pool. A genomic library of 300–400 bp in size was generated and sequenced on a BGISeq500 machine, following the sequencing provider's protocol. Paired‐end sequencing with reads of 150 bp in length was conducted, resulting in approximately 48.3 Gb of sequenced reads.

### Additional whole genome sequencing datasets from Netherland dwarf rabbits and other breeds

Twenty‐four additional whole‐genome sequencing datasets from rabbits were obtained from Carneiro et al. (2014, 2017) (Table S2). These data were generated using a DNA‐pool sequencing approach similar to the one employed in the present study. The DNA pools consisted of DNA from 10 to 20 rabbits each. They included both wild rabbits (14 pools) and domesticated (10 pools) breeds, such as the Belgian Hare, Champagne d’Argent, Dutch, Flemish Giant, French Lop, New Zealand White and Netherland Dwarf breeds. Specifically for the Netherland Dwarf breed, five DNA pools were analysed: two pools composed of individuals homozygous (*dw*/*dw*) for the dwarf allele and three pools composed of individuals heterozygous for the dwarf allele (*Dw*/*dw*). Further details on these data can be found in previous studies (Carneiro et al., 2014, 2017).

### Read mapping and variant detection

Reads were mapped to *Oryctolagus cuniculus* mOryCun1.1 reference genome (GCF_964237555.1) assembled in 2024, which improves the OryCun2.0 genome version assembled in 2009. Read mapping was conducted using the bwa‐mem tool version 0.7.17 (Li & Durbin, 2009) with default parameters. picard v.2.1.1 (https://broadinstitute.github.io/picard/) was used to remove duplicated reads. Variants were called using gatk4 (Poplin et al., 2017), haplotypecaller (indel‐size‐to‐eliminate‐in‐ref‐model set to 10) and variantfiltration (hard‐filter; basic filtering thresholds for SNPs and indels as recommended in the manual; only short indels (<50 bp) were retained), respectively. Structural variants were identified using smoove v.0.2.8 (https://github.com/brentp/smoove). Allele frequencies were estimated by counting the number of reads supporting the reference and alternative alleles divided by the total number of reads overlapping a given position.

### Variant annotation and prioritisation

Variants were annotated using the variant effect predictor v.95.0 tool (McLaren et al., 2016) with gene information extracted from the mOryCun1.1 GFF file. In this genome version, chromosomes are named based on size; therefore, they were renamed to match the OryCun2.0 assembly by cross‐mapping the positions of known genes. For prioritisation, we extracted variants contained with regions showing signatures of selection (derived from *F*_ST_ and pooled heterozygosity analyses; see below) that (i) overlapped with exons (including UTRs) and (ii) had an alternative allele frequency >0.45 in Dwarf Lop and/or Netherland Dwarf rabbits.

### Genetic differentiation analyses

The genetic distance between pairs of populations was estimated by calculating the fixation index (*F*_ST_) value for each SNP, following the method described by Karlsson et al. (2007). These *F*_ST_ values were then averaged for each pairwise comparison, resulting in a distance matrix that was utilised for clustering analysis (hard clustering) and principal component analysis (PCA). Only SNPs that were segregating and not fixed in the two populations being compared were included in the *F*_ST_ computation. The pipelines used for these analyses were developed in either python 3.0 or r 4.2.0 (R Core Team, 2018).

### Detection of signatures of selection and annotation of these genomic regions

Signatures of selection were identified using the *F*_ST_ statistic in a window‐based approach (Bovo et al., 2020). Non‐overlapping windows of size 250 kb (size defined following Rubin et al., 2010), totalling 10 441 windows located in assembled chromosomes (1–21; scaffolds excluded), were scanned. *F*_ST_ values were calculated for each SNP and averaged within the window. Only SNPs that were segregating and not fixed in the two populations or groups (defined based on their body size) being compared were included in the *F*_ST_ computation. In each comparison, only windows with more than 200 variants were kept, and windows with an *F*_ST_ value above the 99.5th percentile of the distribution (top 52 windows) were identified as candidate regions for signatures of selection. This set of windows was further reduced to 21 when the most extreme *F*_ST_ values were selected based on the 99.8th percentile. This was done to identify the most relevant genomic regions from all comparative analyses. For variant prioritisation, we considered expanded genomic windows by adding 125 kb to both ends of each candidate region.

The *F*_ST_ analyses were then complemented by computing the pooled heterozygosity (*H*_P_) statistic, a measure of the overall genetic diversity within a population or a group (Bovo et al., 2020). Similar to *F*_ST_, the *H*_P_ was calculated for each non‐overlapping window using the formula described by Rubin et al. (2010), and extreme windows were selected based on percentile thresholds as defined for the *F*_ST_ analyses (top 52 and 21 windows).

The relevance of genes annotated in all these windows was assessed through a comparative evaluation of human phenotype‐gene relationships extracted from different resources, including: (i) the genome‐wide association study (GWAS) catalogue (GWAS Catalog; Buniello et al., 2019), prioritising associations in humans with ‘height’ as a relevant phenotype; (ii) the UniProt database (The Uniprot Consortium, 2025), considering a collection of 221 proteins related to dwarfism (UniProt keyword: KW‐0242); and (iii) the scientific literature. Most of the GWAS Catalog genes associated with human height, which is relevant for identifying candidate genes associated with body size, derive from the human genomic map released by Yengo et al. (2022) that included more than 5.4 million humans in an association study with height. Pipelines were developed in either python 3.0 or r 4.2.0 (R Core Team, 2018).

Gene enrichment analysis across three different human ontologies was carried out with Enrichr via Fisher's exact test (Chen et al., 2013). The first one, which is the Gene Ontology (GO) Biological Process branch (The Gene Ontology Consortium et al., 2023), was used to evaluate general biological functions of genes. The other two, which are the MGI Mammalian Phenotype (Baldarelli et al., 2024) and DisGeNET (Piñero et al., 2020), were adopted to evaluate genes in the context of human complex traits and diseases. All the genes located in the sweep regions (99.5th percentile) were used as the input set. Terms presenting a Benjamini–Hochberg adjusted *p*‐value (*P*_BH_) < 0.05 were defined as statistically overrepresented.

## RESULTS

### Genotyping results for Dwarf Lop rabbits

Individual genotyping of the *HMGA2* alleles using PCR reported that two out of the 14 Dwarf Lop rabbits used to construct the breed‐specific DNA pool were carriers of the deleted allele at the *HMGA2* gene and were classified to have the *Dw*/*dw* genotype. All the remaining Dwarf Lop rabbits were homozygous for the wild‐type allele and were classified as having the *Dw*/*Dw* genotype. These results indicate that in some dwarf breeds the *dw* allele may segregate at low frequency.

### Genetic differentiation among breeds and wild populations

The sequenced DNA pool of Dwarf Lop rabbits produced approximately 161 million 150 bp reads, resulting in a depth of sequencing of about 17×. Data from all other rabbit DNA pools (Carneiro et al., 2014, 2017) had a depth of sequencing ranging from 6 to 13×. Summary statistics on the sequenced DNA pools are reported in Table S2.

**FIGURE 2:**
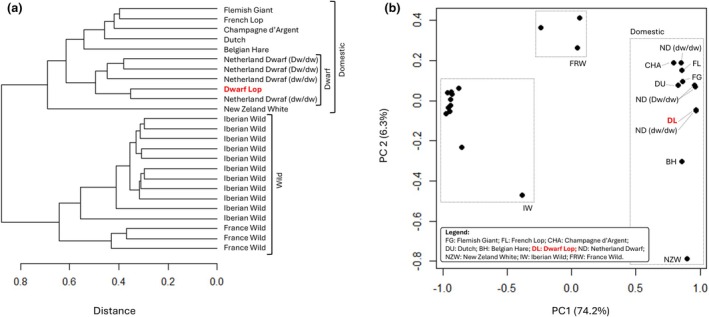
Genetic relationships among rabbit breeds and wild populations. (a) Results of the unsupervised hierarchical clustering analysis; (b) Results of the principal component analysis.

The *F*_ST_ index was used to evaluate the genetic differentiation among pairs of rabbit populations (Figure 2). Clustering analysis based on these pairwise comparisons (Figure 2a) revealed two main branches (and clusters) dividing domesticated rabbits from wild rabbits. Wild rabbits were further divided into two sub‐branches, identifying France and Iberian rabbits, as expected from Carneiro et al. (2014). This clustering into sub‐populations was also supported by PCA (Figure 2b): PC1 separated domesticated and wild populations and accounted for the majority of the variance in the data (74.2%). Cluster analysis also showed that all DNA pools from dwarf rabbits, including the Dwarf Lop breed, clustered together and were distinct from all other domesticated breeds (Figure 2a).

### *F*_ST_ analyses

**FIGURE 3:**
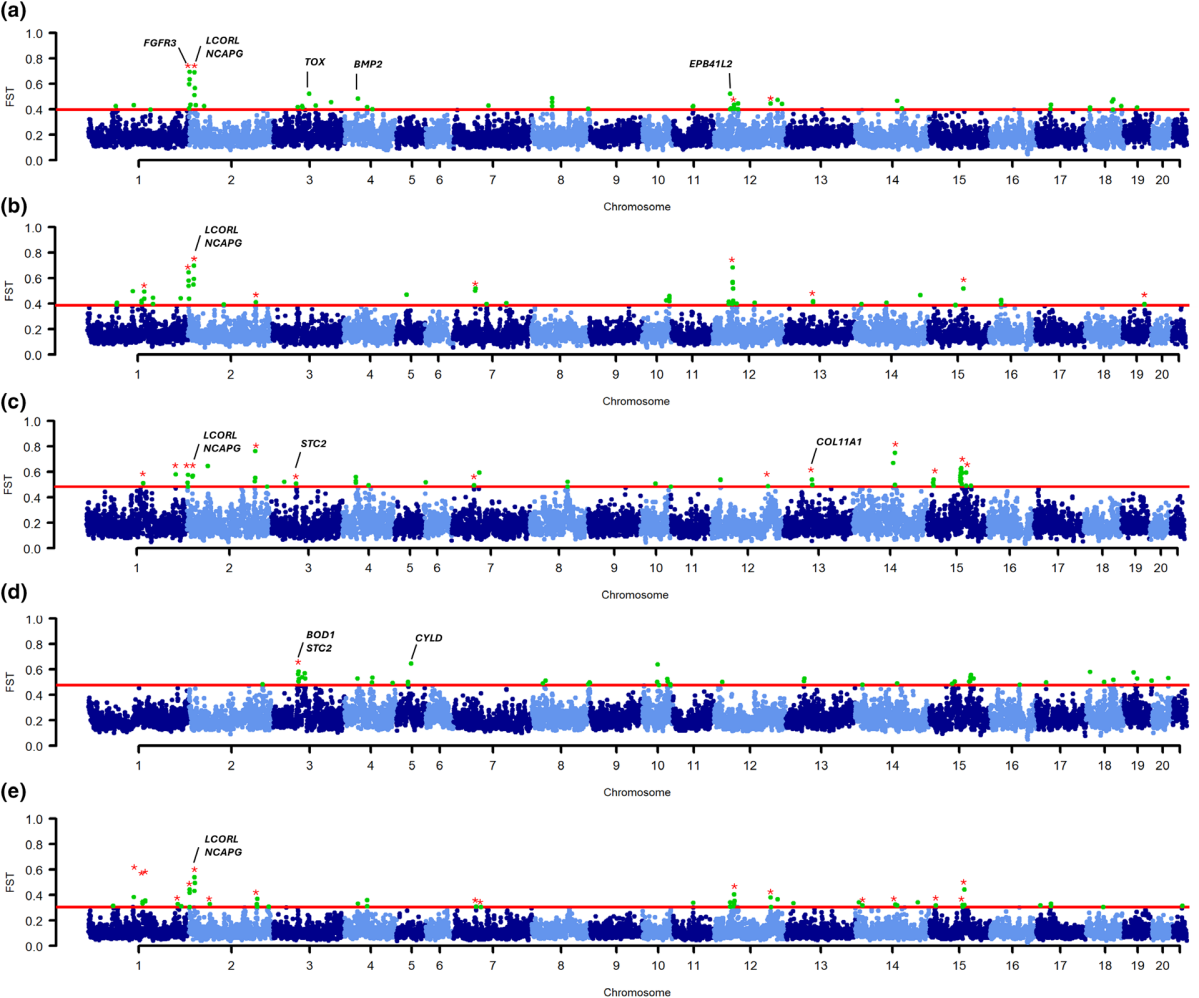
Manhattan plots summarising window‐based *F*_ST_ values across different comparisons: (a) Dwarf Lop breed against other non‐dwarf domesticated breeds grouped together (Belgian Hare, Champagne d’Argent, Dutch, Flemish Giant, French Lop, New Zealand White); (b) Netherland Dwarf rabbits with genotype *Dw*/*dw* against the same non‐dwarf breeds; (c) Netherland Dwarf rabbits with genotype *dw*/*dw* against the same non‐dwarf breeds; (d) Dwarf Lop against Netherland Dwarf rabbits (with genotype *Dw*/*dw* and *dw*/*dw*); and (e) all dwarf rabbit breeds (Dwarf Lop and the Netherland Dwarf rabbits with genotype *Dw*/*dw* and *dw*/*dw*) against all other non‐dwarf domesticated breeds. The red line indicates the threshold in *F*_ST_ used to highlight signatures of selection (99.5th percentile). Green dots represent outlier genomic windows that exceed the defined threshold. The red star symbol (*) highlights regions with annotated genes reported by Carneiro et al. (2017).

Figure 3 displays the Manhattan plots obtained from the *F*_ST_ analyses. We initially contrasted the Dwarf Lop breed pool against non‐dwarf domesticated breeds grouped together (Belgian Hare, Champagne d’Argent, Dutch, Flemish Giant, French Lop, New Zealand White) (Figure 3a). For comparative analyses, we also contrasted Netherland Dwarf rabbits with genotype *Dw*/*dw* and genotype *dw*/*dw* obtained from Carneiro et al. (2014, 2017) against the same non‐dwarf breeds (Figure 3b,c). By comparing these results to those obtained for the Dwarf Lop breed, we could identify if signatures of selection for the Netherland Dwarf rabbits were different from those for the Dwarf Lop breed, using a common reference rabbit genome (mOryCun1.1). Furthermore, we contrasted the Dwarf Lop breed DNA pool with the Netherland Dwarf DNA pools (with genotype *Dw*/*dw* and *dw*/*dw*) (Figure 3d) to directly identify differences between the two breeds. Lastly, we contrasted all dwarf rabbit DNA pools grouped together (from the Dwarf Lop breed and the Netherland Dwarf rabbits with genotype *Dw*/*dw* and *dw*/*dw*) against all other non‐dwarf domesticated breeds grouped together (Figure 3e). This contrast helped identify signatures of selection that may differentiate dwarf breeds from normal‐sized breeds. Wild rabbits were not included in these analyses owing to their high level of genetic diversity and strong population structure when compared to domesticated breeds, which could introduce some biases.

The list of the top genomic windows (99.8th percentile) identified in the *F*_ST_ analysis between the Dwarf Lop breed and the other non‐dwarf breeds is reported in Table 1. These windows were located on seven autosomes, including OCU2, OCU3, OCU4, OCU8, OCU12, OCU14 and OCU18 (Figure 3a). Seven out of 21 windows have also been reported by Carneiro et al. (2017) for the Netherland Dwarf rabbits, also confirmed in this new analysis based on the mOryCun1.1 reference genome (Figure 3b,c; Tables S3, S4). This number increased to 10 out of 52 windows considered when using the 99.5th percentile threshold (Table S5). The first highly differentiated genomic region (*F*_ST_ = 0.694), supported by two additional highly differentiated contiguous windows, was on OCU2, ranging from position 0.5 to 1.25 Mb. This large region contains a total of 22 protein‐coding genes. When annotated with information from the GWAS Catalog on genes associated with human height, it was found that 14 of the 22 protein‐coding genes in these windows may be involved in body size. Among these genes, it is worth mentioning the *fibroblast growth factor receptor 3* (*FGFR3*), as mutations in this gene are known to cause dwarfism and achondroplasia in humans (Shiang et al., 1994). Interestingly, this gene was not annotated in the OryCun2.0 rabbit genome, which was used in previous studies on dwarfism (Carneiro et al., 2017).

**TABLE 1:** List of top differentiated genomic windows identified in the *F*_ST_ analyses (99.8th percentile) including the Dwarf Lop breed and all dwarf breeds together compared with various non‐dwarf breeds. The reported genomic windows are those identified only in the Dwarf Lop breed, in the Dwarf Lop breed analysis and in the combined dwarf breed analysis, and those that emerged only when all dwarf rabbit breeds were used in a combined analysis (the complete list of differentiated genomic regions based on the 99.8th percentile is reported in Tables S6 and S7).

OCU^a^	Window (Mb)^b^	*F*_ST_ ^c^	No. of windows^d^	Genes^e^
Dwarf Lop breed vs. non‐dwarf breeds^f^				
2	3.25–3.50	0.437	1	BLOC1S4*, *MAN2B2*, *LOC138848763*, KIAA0232*, *S100P*, *CFAP184*, *LOC138848764*, PPP2R2C*, *LOC100344712*, TBC1D14*
3	84.50–84.75	0.522	1	TOX*
3	136.25–136.50	0.456	1	–
4	31.50–31.75	0.483	1	BMP2*
8	47.50–47.75	0.488	2	–
12	57.00–57.25	0.445	1	*LOC127493249*
12	160.25–160.50	0.444	1	*TFAP2A*, *LOC100357230*
14	99.50–99.75	0.466	1	–
18	61.00–61.25	0.459	1	*LOC108178480*, *VPS26A*, HKDC1*, HK1*, TSPAN15*, SUPV3L1*, SRGN*, *TACR2*
18	64.25–64.50	0.478	1	P4HA1*, ECD*, *DNAJC9*, ANXA7*, *MRPS16*, *NUDT13*, *LOC108175407*, CFAP70*, *LOC100343030*, *FAM149B1*
Dwarf Lop breed and all dwarf rabbit breeds vs. all other non–dwarf breeds^g^				
2	0.50–1.25	0.694; 0.443	3	CFAP99*, *CTBP1*, *FAM193A*, FGFR3*, HAUS3*, LETM1*, *LOC138848421*, *MAEA*, MXD4*, *NAT8L*, NELFA*, *NICOL1*, NKX1‐1*, NSD2*, POLN*, *RNF4*, SLBP*, SPON2*, TACC3*, TMEM129*, *UVSSA*, ZFYVE28*
2	12.75–13.50^§,#^	0.693; 0.541	3	MED28*, *QDPR*, *LAP3*, DCAF16*, NCAPG*, *CLRN2*, LCORL*, FAM184B*
12	37.75–38.00^†^	0.524; 0.343	1	EPB41L2*, AKAP7*
12	47.25–47.75^§^	0.437; 0.405	2	*FABP7*, *SMPDL3A*, PKIB*, *CLVS2*, *HSF2*, *SERINC1*
12	133.50–133.75^§^	0.448; 0.380	1	CLPSL1*, CLPSL2*, *CLPS*, MAPK14*, FKBP5*, MAPK13*, SLC26A8*, *LHFPL5*, ARMC12*, SRPK1*
12	150.50–150.75	0.475; 0.367	1	*LOC127493240*
All dwarf rabbits breeds vs. all other non‐dwarf domesticated breeds^h^				
1	106.75–107^§^	0.385	1	*LOC100357107*, CADM1*
1	128.00–128.25^§,#^	0.345	1	*SPATA19*, NCAPD3*, *IGSF9B*, *JAM3*
1	134.25–134.50^§,#^	0.358	2	*LOC108178954*, *LOC100353975*, *LOC100353727*, *UBQLN1*, IDNK*, FRMD3*, *LOC100353215*
2	161.25–161.50	0.370	1	–
4	53.50–53.75	0.360	1	CERS5*, FAM186A*, LIMA1*, DIP2B*, ASIC1*, ATF1*, *GPD1*, *SMARCD1*, *COX14*
11	47.75–48.00	0.340	1	*LOC127492839*, CDH18*
13	17.75–18.00	0.335	1	*LOC138850276*
14	8.50–8.75	0.342	1	MORC3*, *LOC100348276*, CLDN14*, *LOC100345975*, DOP1B*, CHAF1B*, *LOC138843083*, *LOC138842983*, *LOC100345716*
14	148.00–148.25	0.344	1	*ACKR4*, ACAD11*
15	82.75–83.00^§^	0.442	1	*LOC127483188*, *LOC127483186*, *THAP9*, *COPS4*, SCD5*, *PLAC8*, *LIN54*, SEC31A*, *COQ2*

^a^ Rabbit chromosome.

^b^ Position in Mb in the *Oryctolagus cuniculus* mOryCun1.1 reference genome (GCF_964237555.1): ^§^genomic windows also identified by Carneiro et al. (2017); ^#^genomic windows also identified by Ballan et al. (2022); ^†^genomic window also identified with pooled heterozygosity analysis in Dwarf Lop rabbits.

^c^ *F*_ST_ value. When two values are presented, the first refers to the ‘*Dwarf Lop breed against other non‐dwarf domesticated breeds*’ comparison whereas the second refers to the ‘*All dwarf rabbit breeds against all other non‐dwarf domesticated breeds*’ comparison.

^d^ Number of consecutive windows.

^e^ Genes annotated in the extended window (**±**125 kb); *Genes association with height reported in the GWAS catalogue.

^f^ Genomic windows identified only in the contrast between the Dwarf Lop breed and all non‐dwarf breeds.

^g^ Genomic windows identified both in the contrast between the Dwarf Lop breed and all non‐dwarf breeds as well as between all dwarf breeds and all non‐dwarf breeds.

^h^ Genomic windows identified only in the contrast between all dwarf breeds and all non‐dwarf breeds.

The second highest differentiated region (*F*_ST_ = 0.693), also supported by two other contiguous genomic windows, is located on OCU2, spanning from 12.75 to 13.50 Mb (considering all three windows). This region includes the *ligand dependent nuclear receptor corepressor like* (*LCORL*) and *non‐SMC condensin I complex subunit G* (*NCAPG*) genes, which have been frequently associated with stature and body size in several species (e.g. Pryce et al., 2011; Takasuga, 2016).

On OCU3, the differentiated region includes the *thymocyte selection associated high mobility group box* (*TOX*) gene, whose function is seemingly unrelated to body size even though the GWAS Catalog reports a highly significant association with human height for this gene. It is worth mentioning that this region is about 3 Mb from the *PLAG1 Zinc Finger* (*PLAG1*) gene, which is well known to influence height and body size in mammals (Karim et al., 2011).

Another differentiated region not previously identified by Carneiro et al. (2017) was on OCU4 (*F*_ST_ = 0.483). This region includes the *bone morphogenetic protein 2* (*BMP2*) gene which encodes a potent inducer of bone and cartilage formation and is annotated as a relevant dwarfism‐related protein in the UniProt database.

Six distinct differentiated regions were identified on OCU12, four of which were not previously identified by Carneiro et al. (2017). All of these regions contained genes that were reported to be strongly associated with human height in the GWAS Catalog (Table 1), although the specific functions influencing body size/dimensions have not yet been characterised for some of them. For example, the region from 37.75 to 38.00 Mb includes the *erythrocyte membrane protein band 4.1 like 2* (*EPB41L2*) gene, which encodes a protein involved in cytoskeletal binding and is among the genes most strongly associated with human height (Yengo et al., 2022), despite its function having no obvious link to body size or dimension. The remaining genomic windows within the 99.8th percentile that contain annotated genes are listed in Table 1.

The results of the contrast between Dwarf Lop rabbits and Netherland Dwarf rabbits are reported in Figure 3d and Table S6. Signatures of selection (99.8th percentile) were identified on eight autosomes (OCU3, OCU4, OCU5, OCU10, OCU13, OCU18, OCU19 and OCU20). We did not observe any overlap of outlier windows between this comparison and the previous one, when we considered the 99.8th percentile. Four regions emerged when we considered the 99.5th percentile (OCU3:68.50–68.75 Mb; OCU4:66.5–66.75 Mb; OCU18:64.25–64.50 Mb; and OCU19:31.25–31.50 Mb; Tables S5 and S6). Most of the identified regions included genes associated with human height (Table S6). The most differentiated region was on OCU5, position 34.25–34.50 Mb (*F*_ST_ = 0.646), where the *CYLD lysine 63 deubiquitinase* (*CYLD*) gene is located. This gene encodes a cytoplasmic protein involved in determining short stature and body mass loss (Cao et al., 2023). In the second top differentiated region on OCU10, no annotated gene is present. The third top differentiated region, located on OCU3 from 60.00 to 60.25 Mb, includes the *biorientation of chromosomes in cell division 1* (*BOD1*) gene, which encodes a protein involved in chromosome biorientation during mitotic division and has been associated with short stature (Hamdan et al., 2020). Additionally, the region OCU3:59.75–60.00 Mb included the *stanniocalcin 2* (*STC2*) gene, previously reported by Carneiro et al. (2017) as a differentiating gene in the comparison between Netherland Dwarf and normal‐sized rabbit breeds also emerged in this comparison. This suggests that markers in this gene, known to affect body size and growth in other mammals (Chang et al., 2008; Gagliardi et al., 2005; Rimbault et al., 2013), may be specific to the Netherland Dwarf rabbits.

The results of the comparison between all dwarf rabbit breeds and all other domesticated breeds are reported in Figure 3e, Table 1, and Table S7. The 99.8th percentile signatures of selection were identified on eight autosomes (OCU1, OCU2, OCU4, OCU11, OCU12, OCU13, OCU14, and OCU15), confirming many regions already identified by Carneiro et al. (2017), with a few novel ones with only eight out of 21 novel regions emerging in this combined analysis. Several of these regions were also identified by Ballan et al. (2022), who compared dwarf and small breeds against normal‐sized and giant breeds using SNP chip data (Table S7). Considering all 99.5th percentile regions, a total of 85 annotated genes were identified, 34 of which have been associated with human height (Table S7). The two most differentiated regions were located on OCU2 and contained the *NCAPG* and *LCORL* genes, reinforcing their relevance in the genetic determination of body size in rabbits.

### *H*_P_ analyses

**FIGURE 4:**
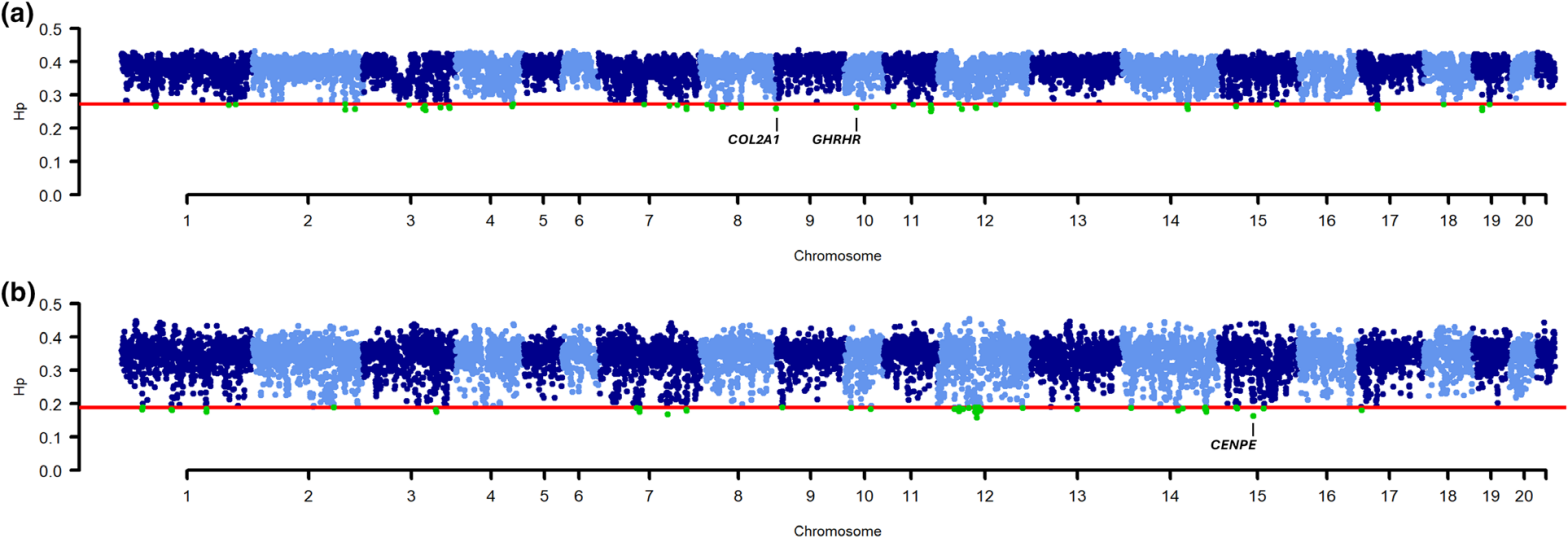
Manhattan plots summarising within‐breed pooled heterozygosity (*H*_P_) in (a) Dwarf Lop and (b) Netherland Dwarf (*Dw*/*dw* and *dw*/*dw*) breeds. The red line indicates the threshold in *H*_P_ used to highlight signatures of selection. Green dots represent outlier genomic windows that exceed the defined threshold.

The *F*_ST_ analyses were complemented with within‐breed pooled heterozygosity (*H*_P_) analyses of Dwarf Lop and Netherland Dwarf (*Dw*/*dw* and *dw*/*dw*) breeds. The Manhattan plots derived from these analyses are presented in Figure 4, and the complete list of outlier genomic windows is provided in Table S8.

In the Dwarf Lop rabbits, the most significant *H*_P_ windows were identified on 10 autosomes (OCU2, OCU3, OCU7, OCU8, OCU10, OCU11, OCU12, OCU14, OCU17 and OCU19). A relevant signal was on OCU8, at position 13.75–13.77 Mb, encompassing *collagen type II alpha 1 chain* (*COL2A1*), a gene linked to type II collagenopathies characterised by skeletal alterations and various forms of dwarfism in humans and cattle (Daetwyler et al., 2014; Deng et al., 2016). This region also showed strong differentiation in the *F*_ST_ comparison between Dwarf Lop and Netherland Dwarf breeds (Table S6). Another important signal was observed on OCU10, at position 22.25–22.250 Mb, encompassing the *growth hormone‐releasing hormone receptor* (*GHRHR*) gene. Mutations in this gene cause another form of human dwarfism (Salvatori et al., 1999). When examining all 52 windows, *H*_P_ signals on OCU8, OCU12, OCU15 and OCU17 overlapped with those identified by *F*_ST_ analyses (Table S8).

In the Netherlands Dwarf population, the 21 most extreme *H*_P_ windows were distributed across seven chromosomes (OCU1, OCU3, OCU7, OCU12, OCU14, OCU15 and OCU19). Among them, the signal on OCU15, at position 62.00–62.25 Mb, encompassed the *centromere protein E* (*CENPE*) gene, which is linked to microcephalic primordial dwarfism, a human disorder characterised by severe growth retardation and skeletal dysplasia (Khetarpal et al., 2016). Out of the full set of 52 candidate windows, *H*_P_ signals on OCU2 and OCU15 overlapped with those identified by *F*_ST_ analyses (Table S8).

A few genomic regions overlapped or were located <1 Mb of each other between the two breeds. These regions were only found on OCU7 and OCU12, where no obvious candidate genes related to growth or height were detected.

### Uncovering relevant variants in differentiated gene regions for dwarf rabbits

Annotation and prioritisation of variant in genes detected by *F*_ST_ analyses resulted in 3342 SNPs and 374 short insertions/deletions (indels) overlapping exons of 239 genes, included in the extended signatures of selection regions identified in the comparisons between Dwarf Lop rabbits and the non‐dwarf breeds, as well as all dwarf DNA pools against the other breeds. A few variants were present exclusively in either Dwarf Lop rabbits or in all dwarf rabbits (Table S9). These included a total of 68 SNPs and 48 InDels affecting 47 genes. Shared by all dwarf rabbits, we identified: (i) five variants in the 3′‐UTR of the genes *NELL2 interacting cell ontogeny regulator 1* (*NICOL1*), *DNA polymerase nu* (*POLN*), *zinc finger FYVE‐type containing 28* (*ZFYVE28*), *Wolframin ER transmembrane glycoprotein* (*WFS1*); (ii) one frameshift variant in the gene (*DDB1 and CUL4 associated factor 16*) *DCAF16*; and (iii) two non‐coding transcript variants in the genes LOC103350447 and LOC138849597 (Table S9).

It is interesting to note that *NICOL1*, *POLN* and *ZFYVE28* are in the OCU2 regions that include genes involved in the Wolf–Hirschhorn Syndrome (WHS), a rare human disorder characterised by a typical facial appearance called Greek warrior helmet facies, intellectual disability and growth delay, among others (Paradowska‐Stolarz, 2014). *WFS1* is linked to Wolfram Syndrome 1 (WS1), a severe neurodegenerative disorder that includes short stature as one of its clinical manifestations (Rigoli et al., 2018). The gene *DCAF16* is in the same differentiated region of OCU2 that includes *NCAPG* and *LCORL*. *DCAF16* is highly significantly associated with human height. In particular, the 1 bp insertion led to the creation of a premature stop codon, resulting in a truncated protein at position Ser23 (XP_069924194.1:p.Ser23Ter) out of the 216 residues (Table S8). Focusing on variants that were private and fixed in Dwarf Lop rabbits, 15 were found to affect the protein‐coding regions of the following genes: *NICOL1*, *KIAA0232*, *LCORL*, *KICSTOR subunit 2* (*KICS2*) and *ecdysoneless cell cycle regulator* (*ECD*) (Table S9). All the other variants were located either in the 5′‐or 3′‐UTR regions. The 12.1 kb deletion at the *HMGA2* locus described by Carneiro et al. (2017) was identified in Netherland Dwarf rabbits, confirming the results obtained using the OryCun2.0 genome version (Carneiro et al., 2017). Manual inspection of sequenced reads derived from the Dwarf Lop DNA pool to the *HMGA2* gene regions identified the presence of a few reads that defined the deletion in this gene, confirming the genotyping results. Additionally, the Dwarf Lop DNA pool indicated the presence of an inversion of 126 nt (NC_091442.1:g.68181442_68181568inv) in the fourth intron of the *HMGA2* gene, present at a high frequency (0.67), which may represent a case of genetic heterogeneity at this gene.

From the evaluation of *H*_P_ signals, a total of 78 SNPs and 30 short indels were exclusively identified in either Dwarf Lop or Netherland Dwarf rabbits, overlapping exons of 34 genes (Table S10). Among this set of variants, none were found in the previously discussed candidate genes (*CENPE*, *GHRHR*, *COL1A1*). The remaining identified variants were found in genes that are not currently known to be associated with size variation or dwarf phenotypes.

### Functional enrichment analysis of genes in signatures of selection regions

To identify functionally enriched biological processes, we analysed the 176 genes located within the 99.5th percentile selective sweep regions identified in the *F*_ST_ comparison between Dwarf Lop rabbits and all other non‐dwarf domesticated breeds (Table S5). Significant results (*P*_BH_ <0.05) were observed when considering the MGI Mammalian Phenotype functional sets, which identified terms such as ‘increased cranium width’ (MP:0000441) and ‘abnormal foramen magnum morphology’ (MP:001094) (Table S11). These terms included the *fibroblast growth factor receptor 2* (*FGFR2*; OCU18), *FGFR3* (OCU2), *alpha‐l‐iduronidase* (*IDUA*: OCU2) and *laminin subunit alpha 2* (*LAMA2*; OCU12) genes. When looking at the DisGeNET libraries, we found several relevant terms linked to specific characteristics of the Dwarf Lop breed (Table S11), particularly in skeletal‐related functions. These included ‘Wolf–Hirschhorn Syndrome’ (*P*_BH_ = 1.10 × 10^−10^), primarily listing genes located on the top differentiated regions on OCU2 (*leucine zipper and EF‐hand containing transmembrane protein 1*, *LETM1*; *stem‐loop histone mRNA binding protein*, *SLBP*; *C‐terminal binding protein 1*, *CTBP1*; *nuclear receptor binding SET domain protein 2*, *NSD2*; *negative elongation factor complex member A*, *NELFA*; *complexin 1*, *CPLX1*; *FGFR3*, and *fibroblast growth factor receptor like 1*, *FGFRL1*; mapped from positions 0.25 to 1.25 Mb; and *leucine aminopeptidase 3*, *LAP3*, from 12.75 to 13.00 Mb), ‘abnormal sternal ossification’ (*P*_BH_ = 3.11 × 10^−07^), ‘rib segmentation abnormalities’ (*P*_BH_ = 3.11 × 10^−07^), ‘craniofacial asymmetry’ (*P*_BH_ = 8.40 × 10^−07^), and ‘Pitt–Rogers–Danks Syndrome’ (*P*_BH_ = 1.50 × 10^−06^), among others (Table S11). Wolf–Hirschhorn Syndrome is characterised by symptoms like mental retardation, growth retardation and craniofacial dysmorphisms. Pitt–Rogers–Danks syndrome is considered part of the clinical spectrum of Wolf–Hirschhorn Syndrome. Although no GO terms reached Benjamini–Hochberg adjusted significance, the two top terms identified are linked to body size and skeletal development (Table S11): bone morphogenesis (GO:0060349) and bone development (GO:0060348) had gene sets with a raw *p* < 0.0005 (Table S11). These sets included the *FGFR2* (OCU18), *FGFR3* (OCU2), *transcription factor AP‐2 alpha* (*TFAP2A*; OCU2) and *osteoglycin* (*OGN*; OCU1) genes.

When we analysed the 178 genes located within the 99.5th percentile selective sweep regions identified in the comparison between all dwarf rabbit breeds and all other non‐dwarf domesticated breeds, we obtained broadly similar results, particularly with respect to general macro‐level biological processes, to those obtained in the comparison between the Dwarf Lop breed and the normal‐sized breeds reported above (Table S12). The results were marginally significant (*P*_BH_ <0.10) when considering the MGI Mammalian Phenotype functional sets, which identified terms related to vertebrae morphology. When we considered the DisGeNET libraries, the most significant term was ‘Wolf–Hirschhorn Syndrome’, already identified in the previous comparison. Again, no GO terms reached Benjamini–Hochberg adjusted significance. However, the top GO term identified ‘Embryonic Skeletal System Morphogenesis (GO:004804)’ as one of the most relevant processes (Table S12). The enrichment analyses of genes located in *H*_P_ regions did not result in any significant terms.

## DISCUSSION

This study provided an overview of the genetic background influencing the small size of dwarf rabbits. We used whole genome resequencing data from DNA pools of dwarf and non‐dwarf rabbit breeds, and through two complementary genome‐wide approaches, *F*_ST_ and *H*_P_ analyses, identified several major loci that differentiated these two groups defined according to body size, and defined some genetic peculiarities of dwarf breeds.

Despite morphological differences between Dwarf Lop and Netherland Dwarf breeds, mainly in craniofacial shape and ear position and shape, these breeds are genetically closer to each other than to any of the non‐dwarf breeds. These findings are consistent with previous results based on SNP chip genotyping data, which included the Coloured Dwarf breed (a breed with similar characteristics to the Netherland Dwarf breed) and the Dwarf Lop breed (Ballan et al., 2022, 2023). This indirectly indicates that small body size in dwarf rabbit breeds may share a partially common genetic background.

An important difference between the Dwarf Lop breed and the Netherland Dwarf breed lies in the lower frequency of carriers of the deleted allele at the *HMGA2* gene in the former breed, compared to the Netherland Dwarf breed, where most animals seem to be heterozygous (Carneiro et al., 2017). Since Dwarf Lop rabbits included in this study were randomly sampled, these results may provide an initial estimation of the frequency of the deleted *HMGA2* allele in this breed (approximately 7%). The deleted *HMGA2* allele also segregates in other dwarf rabbit populations, where not all animals are carriers of this variant (Nguyen et al., 2024), further suggesting that other loci may contribute to determining small size in rabbits.

Oligogenic or polygenic contributions to the body size of animals have been consistently reported in other domestic species, including dogs, cattle, horses and chickens (Boyko et al., 2010; Lyu et al., 2023; Makvandi‐Nejad et al., 2012; Rimbault et al., 2013; Wang et al., 2024; Wu et al., 2021). This is confirmed by the results of the *F*_ST_ and *H*_P_ analyses that we conducted in rabbits, which further extend what was reported by Carneiro et al. (2017). A polygenic contribution with a few major genes also agrees with early studies in rabbits that investigated the genetic basis of size inheritance using controlled crosses (Castle, 1914, 1929; MacDowell, 1914a, 1914b; Pease, 1928; Punnett & Bailey, 1918; Wright, 1918).

Several shared genomic regions emerged when comparing the results from analyses contrasting Dwarf Lop and Netherland Dwarf breeds individually with non‐dwarf breeds alone, as well as when both dwarf breeds were combined and compared collectively against other breeds. The combination of Dwarf Lop and Netherland Dwarf breeds further strengthened the contrasting power against non‐dwarf breeds. The *F*_ST_ analysis of the combined dwarf breeds revealed an additional 38% or 47% differentiated genomic windows compared with just using Netherland Dwarf DNA pools, considering the 99.8th and 99.5th percentiles, respectively (Table S7). A few other regions emerged with the *H*_P_ analyses.

Among the most relevant shared *F*_ST_‐detected regions, OCU2 genomic windows containing *LCORL*/*NCAPG* emerged consistently in both individual breed analyses and in the combined dwarf breeds comparison, also confirming results obtained with high‐density SNP data analyses (Ballan et al., 2022). The *LCORL*/*NCAPG* region has been consistently associated with variability in body size, weight, and stature in many different species such as humans, cattle, dogs, horses, pigs, sheep and chickens (Al‐Mamun et al., 2015; Bai et al., 2025; Bovo et al., 2021; Pryce et al., 2011; Rubin et al., 2012; Sahana et al., 2015; Tetens et al., 2013). Sequencing data for the Dwarf Lop DNA pool revealed several high‐frequency variants in this breed affecting the protein structure of the *LCORL* gene and a frameshift mutation in the *DCAF16* gene, located in the same genomic window. To determine any potential causative effects of these variants, further evaluation is needed of the linkage disequilibrium among the identified polymorphisms in this region, their allele frequencies in other rabbit breeds, and their functional roles. Another independent region on OCU2 emerged by using the latest version of the reference rabbit genome and contains the *FGFR3* gene, which encodes a member of the fibroblast growth factor receptor that plays a role in bone development and maintenance. Mutations in this gene cause achondroplasia, which is the most common form of human dwarfism (Di Rocco et al., 2014; Shiang et al., 1994) and hereditary chondrodysplasia in sheep (Beever et al., 2006). By analysing the sequencing data obtained from the rabbit DNA pools we did not identify any private mutations within this gene for dwarf rabbits, suggesting that non‐fixed regulatory mutations at this gene may potentially contribute to body size variation in rabbits.

Some differences between Dwarf Lop and Netherland Dwarf breeds have emerged. Most of these differentiated genomic regions contain genes that have already been associated with body size or height in other species. For example, the region on OCU5 containing the *CYLD* gene seems specifically different in Dwarf Lop rabbits. A study based on *Cyld* knockout mice has demonstrated that this gene plays an important role in osteogenesis and bone structure and that loss of function mutations may determine short stature and reduced body mass (Cao et al., 2023). Another region containing the *STC2* gene appeared to be specifically differentiated in the Netherland Dwarf breed. This gene encodes a secreted glycoprotein, expressed in many tissues, with an autocrine or paracrine role acting as a negative pre‐ and post‐natal growth regulator (Chang et al., 2008; Gagliardi et al., 2005; Jepsen et al., 2015). This gene has been associated with body size and height in humans, cattle and dogs (Bai et al., 2025; Boyko et al., 2010; Marouli et al., 2017; Rimbault et al., 2013).

When comparing the *H*_P_ results, additional differences between the Dwarf Lop and Netherland Dwarf breeds were identified. For example, two genomic regions on OCU8 and OCU10, containing two relevant candidate genes, were found in the Dwarf Lop breed. Another genomic region on OCU15, which included another interesting candidate gene, was discovered in the Netherland Dwarf breed. In the OryCun2.0 rabbit genome version, the region assigned to OCU8 in the mOryCun1.1 reference genome was located in an unassembled scaffold (Un0251). This region was previously identified as a selective sweep in Dwarf Lop rabbits in a study using high‐density SNP chip data (Ballan et al., 2022), further confirming the results obtained in the current study. The region contains the *COL2A1* gene, mutations in which have been shown to cause various skeletal defects in mammals, such as achondrogenesis type II in humans (Forzano et al., 2007; Körkkö et al., 2000) and bulldog‐type dwarfism in cattle (Daetwyler et al., 2014; Häfliger et al., 2019). The OCU10 region contains the *GHRHR* gene, which encodes a receptor for growth hormone‐releasing hormone. Binding of this hormone to the receptor is a pivotal step in the regulation of growth hormone synthesis and release. A missense mutation in the *Ghrhr* gene is responsible for the little locus in mice, characterised by reduced growth hormone secretion and a dwarf phenotype (Godfrey et al., 1993). Mutations in this gene in humans have been associated with isolated growth hormone deficiency, leading to a type of dwarfism similar to the *little* phenotype in mice (Cohen et al., 2019; Salvatori et al., 1999; Wajnrajch et al., 1996). The candidate gene located on OCU15 is *CENPE*, which encodes a core kinetochore component that accumulates in the G2 phase of the cell cycle. Mutations in this gene are responsible for a form of microcephalic primordial dwarfism in humans (Khetarpal et al., 2016; Mirzaa et al., 2014), which bears some resemblance to the craniofacial peculiar morphology of Netherland Dwarf rabbits.

The results of functional enrichment analysis revealed that genes included in the top differentiated regions, whether considering only one dwarf breed or both breeds, may be involved in skeletal‐related structures or abnormalities, depending on the functional sets. This further highlights that the small size of dwarf rabbits may be due to body structural reduction mechanisms that involve many genes. It is also worth noting that a recurrent term was ‘Wolf–Hirschhorn Syndrome’. This condition is characterised by growth reduction and craniofacial alterations, common characteristics of the two dwarf breeds investigated. Since the rabbit is often used as a biomedical model for many biological functions and clinical aspects (Miller et al., 2014), dwarf breeds may offer valuable opportunities for studying altered growth, as well as skeletal functions and structures.

Dwarf breeds are mainly considered fancy breeds. In some of these breeds, fancy breeders have introgressed some genetic characteristics from other breeds that define specific traits, such as the lop ears in Dwarf Lop. Other introgressed genetic characteristics from non‐dwarf breeds determine the variability in coat colours found in the dwarf populations. Since coat colour is not a fixed trait in the analysed dwarf breeds, the *F*_ST_ analysis did not reveal any signatures of selection including major genes affecting this external feature. Therefore, breeding practices that introgressed different coat colour alleles in dwarf breeds may have influenced the polygenic patterns of small size. Coat colour alleles are important genetic characteristics of many rabbit breeds (Fontanesi, 2021a). After introgressing mutations that determine specific coat colours from other normal‐sized breeds, breeders had to once again select for the small size of the animals. This may have led to an increase in the number of loci with relatively small effects on body size. The traditional use of dwarf rabbit breeds as pets worldwide may have contributed to increasing genetic variability within breeds and populations, especially since not all dwarf rabbits are registered in national herd books and do not have a specific standard. Some rabbits indicated to be dwarf individuals by pet breeders may actually be better described as animals with small size, enlarging the range of body dimensions in these populations. However, some major loci remained, including alleles that determine monogenic forms of dwarfism, such as the deleted *HMGA2* form. Furthermore, it would be interesting to clarify if other monogenic forms of dwarfism, described in the early rabbit genetic literature (reviewed in Fontanesi, 2021b), are still segregating in rabbit breed populations and if they can be considered true monogenic determinants of small size in this species.

## AUTHOR CONTRIBUTIONS

**Samuele Bovo:** Conceptualization; methodology; investigation; visualization; data curation; writing – original draft; writing – review and editing; formal analysis. **Miguel Carneiro:** Conceptualization; methodology; investigation; validation; writing – review and editing; writing – original draft; resources; funding acquisition. **Anisa Ribani:** Investigation; writing – review and editing; data curation; validation. **Matteo Bolner:** Investigation; validation; formal analysis; writing – review and editing. **Valeria Taurisano:** Validation; formal analysis; investigation; writing – review and editing. **Giuseppina Schiavo:** Investigation; methodology; formal analysis; data curation; software. **Michele Schiavitto:** Resources; writing – review and editing; funding acquisition. **Francesca Bertolini:** Conceptualization; investigation; methodology; writing – review and editing. **Luca Fontanesi:** Conceptualization; investigation; funding acquisition; writing – original draft; writing – review and editing; validation; project administration; resources; supervision; methodology.

## FUNDING INFORMATION

This study was funded by the PSRN (Programma di Sviluppo Rurale Nazionale) Cun‐Fu and Cun‐Fu 2 projects (co‐funded by the European Agricultural Fund for Rural Development of the European Union and by the Italian Ministry of Agriculture, Food Sovereignty and Forestry ‐ MASAF) and by the University of Bologna RFO 2024 programme. MC was funded by the Portuguese Foundation for Science and Technology (FCT, https://www.fct.pt) research contract CEECINST/00014/2018/CP1512/CT0002.

## CONFLICT OF INTEREST STATEMENT

The authors declare they do not have any conflict of interests.

## Supporting information


Table S1‐S2.

Table S3‐S12.

## Data Availability

The data generated in this study are included in this published article, in its supplementary information files and are also available on Zenodo at https://doi.org/10.5281/zenodo.15076136. The newly produced whole genome sequencing data are available in the European Nucleotide Archive under the project number PRJEB87433.
