# Supplementary material for: Signatures of selection detected from whole‐genome sequencing indicate that the small body size in dwarf rabbit breeds is caused by polygenic effects with a few major loci
Source: Anim Genet. 2025 Jul 3;56(4):e70025. doi: 10.1111/age.70025 (PMC12224565; doi:10.1111/age.70025)
Supplement: Supplementary file 1 — Table S1‐S2. [file AGE-56-0-s002.docx]

**Supporting information for:**

**Signatures of selection detected from whole-genome sequencing indicate that the small body size in dwarf rabbit breeds is caused by polygenic effects with a few major loci**

Samuele Bovo^1^, Miguel Carneiro^2,3^, Anisa Ribani^1^, Matteo Bolner^1^, Valeria Taurisano^1^, Giuseppina Schiavo^1^, Michele Schiavitto^4^, Francesca Bertolini^1^, Luca Fontanesi^1^

^1^ Animal and Food Genomics Group, Division of Animal Sciences, Department of Agricultural and Food Sciences, University of Bologna, Viale Giuseppe Fanin 46, 40127, Bologna, Italy

^2^ BIOPOLIS Program in Genomics, Biodiversity and Land Planning, CIBIO, Campus de Vairão, Vairão, Portugal

^3^ CIBIO, Centro de Investigação em Biodiversidade e Recursos Genéticos, InBIO Laboratório Associado, Universidade do Porto, Campus de Vairão, Portugal

^4^ Associazione Nazionale Coniglicoltori Italiani (ANCI), Contrada Giancola snc, 71030 Volturara Appula (FG), Italy

* Corresponding author

E-mail addresses:

LF: [luca.fontanesi@unibo.it](mailto:luca.fontanesi@unibo.it)

**Table S1.** PCR primers, PCR conditions and genotyping protocols for the *HMGA2* deleted gene region.

| **Primer pair names^1^** | **Forward and reverse primers (5’-3’)^2^** | **PCR conditions^3^** | **Expected size of the fragments^4^** |
| --- | --- | --- | --- |
| Primer_3_Fwd Primer_4_Rev | CAAAGGGAACAAGCCAATGT GGGGTGCCATTAGGAACTTT | 62 °C | 172 bp (*Dw* allele)  N/A (*dw* allele) |
| Primer_5_Fwd Primer_6_Rev | GGTTCCTAGCTAAGTGTGAGTTCC GCTCACAGCTCCCTCCTAAA | 62 °C | 12,667 bp (*Dw* allele)  518 bp (*dw* allele) |

^1^Primers are from Carneiro et al. (2017). Two primer multiplex PCR was carried out.

^2^The targeted rabbit *HMGA2* regions were: (i) Primer_3_Fwd – intron #1, (ii) Primer_4_Rev – intron #1, (iii) Primer_5_Fwd – upstream, (iv) Primer_6_Rev – intron #2.

^3^Annealing temperature. PCR was carried out in a total reaction volume of 14 µL. The reaction mix contained 2X of the Kapa HiFi HotStart ReadyMix PCR kit (Kapa Biosystems, Boston, Massachusetts, USA), 10-50 ng of template DNA and 4.2 µL of each primer. PCR profile was as follows: 5 min at 95 °C, 45 amplification cycles of 30 sec at 95 °C, 30 sec at the appropriate annealing temperature, 30 sec at 72 °C and a final extension of 5 min at 72 °C. PCR was performed using a SimpliAmp Thermal Cycler (Thermo Fisher Scientific, Waltham, MA, USA).

^4^Expected size (bp) of the fragments is reported for the wild type (*Dw*) and dwarf (*dw*) alleles, respectively. Primer length is included. Genotypes are: (i) dwarf (*Dw*/*dw*) – 172 bp and 518 bp fragments, (ii) peanut (*dw*/*dw*) – 518 bp fragment and (iii) wild type (*Dw*/*Dw*) – 172 bp fragment. Amplified DNA fragments obtained were electrophoresed on 2.5% agarose gels in TAE1X buffer, gels were stained with 1X GelRed Nucleic Acid Gel Stain (Biotium Inc., Hayward, CA, USA).

**Table S2.** Summary statistics for the sequenced DNA pools.

| **ENA project^1^** | **ENA sample^2^** | **Population / Breed^3^** | **DP (x)^4^** | **Coverage (%)^5^** | **Duplication (%)^6^** |
| --- | --- | --- | --- | --- | --- |
| *Domestic* | | | | | |
| PRJEB87433 | ERS23841884 | Dwarf lop | 16.4 | 98.5 | 1.1 |
| PRJNA242290 | SAMN02786082 | Belgian hare | 10.7 | 99.4 | 0.5 |
| PRJNA242290 | SAMN02786075 | Champagne d'argent | 11.2 | 99.5 | 0.5 |
| PRJNA242290 | SAMN02786069 | Dutch | 10.5 | 99.5 | 0.4 |
| PRJNA354575 | SAMN06054243 | Netherland dwarf (Dw/dw) | 5.6 | 98.1 | 1.8 |
| PRJNA354575 | SAMN06054244 | Netherland dwarf (Dw/dw) | 5.7 | 98.1 | 1.8 |
| PRJNA354575 | SAMN06054245 | Netherland dwarf (dw/dw) | 6.5 | 98.8 | 1.3 |
| PRJNA354575 | SAMN06054246 | Netherland dwarf (dw/dw) | 6.6 | 98.8 | 1.3 |
| PRJNA242290 | SAMN02786080 | Flemish giant | 12.7 | 99.5 | 0.5 |
| PRJNA242290 | SAMN02782768 | French lop | 12.3 | 99.5 | 0.6 |
| PRJNA242290 | SAMN02786079 | New Zealand white | 11.6 | 99.3 | 0.5 |
| *Wild* | | | | | |
| PRJNA242290 | SAMN02786072 | Wild French | 11.0 | 99.7 | 0.6 |
| PRJNA242290 | SAMN02786076 | Wild French | 12.4 | 99.8 | 0.6 |
| PRJNA242290 | SAMN02786078 | Wild French | 12.1 | 99.7 | 0.6 |
| PRJNA242290 | SAMN02782771 | Wild Iberian | 11.4 | 99.6 | 0.6 |
| PRJNA242290 | SAMN02782772 | Wild Iberian | 11.8 | 99.6 | 0.6 |
| PRJNA242290 | SAMN02782773 | Wild Iberian | 11.8 | 99.5 | 0.6 |
| PRJNA242290 | SAMN02786068 | Wild Iberian | 11.9 | 99.5 | 0.6 |
| PRJNA242290 | SAMN02786070 | Wild Iberian | 12.5 | 99.5 | 0.6 |
| PRJNA242290 | SAMN02786071 | Wild Iberian | 11.6 | 99.6 | 0.6 |
| PRJNA242290 | SAMN02786073 | Wild Iberian | 12.3 | 99.6 | 0.6 |
| PRJNA242290 | SAMN02786077 | Wild Iberian | 10.7 | 99.5 | 0.5 |
| PRJNA242290 | SAMN02786081 | Wild Iberian | 11.4 | 99.4 | 0.6 |
| PRJNA242290 | SAMN02786083 | Wild Iberian | 12.2 | 99.6 | 0.6 |
| PRJNA242290 | SAMN02786084 | Wild Iberian | 6.4 | 98.8 | 0.4 |

^1^Projects registered with the European Nucleotide Archive (ENA; https://www.ebi.ac.uk/ena/browser/home); ^2^Samples registered with ENA; ^3^ Except for Dwarf lop rabbits, here investigated for the first time in this study, information derives from ENA, Carneiro et al. (2014; 2017); ^4^Depth pf sequencing considering deduplicated reads mapped to the *Oryctolagus cuniculus* mOryCun1.1 reference genome (GCF_964237555.1); ^5^Genome coverage considering deduplicated reads mapped to the *Oryctolagus cuniculus* mOryCun1.1 reference genome (GCF_964237555.1); ^6^Fraction of duplicated reads in the raw alignment data.

**Table S3.** Differentiated genomic regions in Netherland Dwarf (*Dw/dw*) rabbits. This population was compared with other domestic non-dwarf populations grouped together, including Belgian Hare, Champagne d’Argent, Dutch, Flemish Giant, French Lop, New Zealand White.

[Please refer to the spreadsheet file.]

**Table S4.** Differentiated genomic regions in Netherland Dwarf (*dw/dw*) rabbits. This population was compared with other domestic non-dwarf populations grouped together, including Belgian Hare, Champagne d’Argent, Dutch, Flemish Giant, French Lop, New Zealand White.

[Please refer to the spreadsheet file.]

**Table S5.** Differentiated genomic regions in Dwarf Lop rabbits. This population was compared with other domestic non-dwarf populations grouped together, including Belgian Hare, Champagne d’Argent, Dutch, Flemish Giant, French Lop, New Zealand White.

[Please refer to the spreadsheet file.]

**Table S6.** Differentiated genomic regions detected by comparing Dwarf Lop against Netherland Dwarf (*Dw/dw* and *dw/dw*) breeds.

[Please refer to the spreadsheet file.]

**Table S7.** Differentiated genomic regions detected by comparing Dwarf Lop and Netherland Dwarf (*Dw/dw* and *dw/dw*) breeds against all other domestic breeds.

[Please refer to the spreadsheet file.]

**Table S8.** Table S8. Within-breed pooled heterozygosity (HP) in Dwarf Lop and Netherland Dwarf (Dw/dw and dw/dw) breeds.

[Please refer to the spreadsheet file.]

**Table S9.** Results of variant annotation and prioritization for Dwarf Lop sequence information compared with that of other sequence datasets. Investigated regions are those from F_ST_ analyses.

[Please refer to the spreadsheet file.]

**Table S10.** Results of variant annotation and prioritization for Dwarf Lop and Netherland Dwarf sequence information compared with that of other sequence datasets. Investigated regions are those from HP analyses.

[Please refer to the spreadsheet file.]

**Table S11.** Gene enrichment analysis results for genes identified in differentiated genomic regions identified in Dwarf Lop rabbits.

[Please refer to the spreadsheet file.]

**Table S12.** Gene enrichment analysis results for genes identified in differentiated genomic regions identified in dwarf rabbits.

[Please refer to the spreadsheet file.]
